# Prospective case‐control cohort analysis of two‐day/two‐stage pelvic exenteration surgery: Safety, feasibility, acceptability and medium‐term outcomes

**DOI:** 10.1111/codi.70353

**Published:** 2025-12-29

**Authors:** Charles T. West, Yousif Salem, Siddharth Jain, Lewis Matthews, Julian Smith, Marios Nicolaou, Hideaki Yano, Malcolm A. West, Alex H. Mirnezami

**Affiliations:** ^1^ Southampton Complex Cancer and Exenteration Team University Hospital Southampton Southampton UK; ^2^ Department of Academic Surgery, Cancer Sciences University of Southampton Southampton UK; ^3^ The Shackleton Department of Anaesthesia University Hospital Southampton NHS Foundation Trust Southampton UK; ^4^ Urology Department University Hospital Southampton NHS Foundation Trust Southampton UK; ^5^ Department of Plastic and Reconstructive Surgery University Hospital Southampton Southampton UK; ^6^ NIHR Southampton Biomedical Research Centre, Perioperative Medicine and Critical Care Theme University Hospitals Southampton NHS Foundation Trust Southampton UK

**Keywords:** complications, critical care, health‐related quality of life, pelvic exenteration

## Abstract

**Aim:**

Pelvic exenteration (PE) is the only curative option for extensive pelvic cancers. Advances have facilitated increasingly complex resectional and reconstructive components, including per‐operative oncological adjuncts such as intraoperative radiotherapy. Cumulatively, these components increase operative duration beyond what is feasible within a single conventional operating day. Two‐day/two‐stage PE addresses this, but little is known about this approach. This study aims to evaluate the feasibility, safety and medium‐term outcomes of a two‐day/two‐stage PE.

**Method:**

Consecutive patients (2010–2025) from a prospectively maintained high‐volume PE unit database (*n* = 373) undergoing two‐day/two‐stage PE were compared against a matched control cohort of single‐day cases lasting ≥15 h. EQ5D‐5L and decision regret scores were longitudinally collected after 2021. Surgical, oncological and health‐related quality‐of‐life outcomes were evaluated.

**Results:**

Twenty‐seven patients underwent two‐day/two‐stage PE, and 38 had one‐day PE; more anal cancers were in the two‐day/two‐stage group (*p* = 0.012); median follow‐up was 24.2 months. No 90‐day mortalities occurred; 3‐year overall survival was 54.4% for two‐day/two‐stage PE and 70.5% for one‐day PE (*p* = 0.31); and R0‐resection rates were 82% and 76%, respectively (*p* = 0.76). Major morbidity occurred in 56% and 47% (*p* = 0.62), with a median length of stay of 37 and 27 days (*p* = 0.07) and intensive care days of 5 and 3 (*p* = 0.08). 12‐month EQ5D‐5L utility scores were 0.79 and 0.81 (*p* = 0.96), with low 12‐month decision regret in both groups (*p* = 0.15).

**Conclusion:**

Two‐day/two‐stage PE is safe and feasible, potentially representing the only option for highly selected patients needing high‐complexity PE with multiple components. Although equivalent R0‐resections were obtained, medium‐term oncological outcomes were poorer in patients undergoing two‐day/two‐stage interventions.

**What does this paper add to the literature?**

This is the first paper to report on a two‐day/two‐stage pelvic exenteration surgery, demonstrating that it is feasible, safe and accepted by patients. Such an approach may represent the only option for some patients needing a very high‐complexity pelvic exenteration with multiple resectional and reconstructive components. Considering the acceptable oncological outcomes, this may be their only choice—when compared to palliative options characterised by declining quality of life until death.

## INTRODUCTION

Pelvic exenteration (PE) surgery may be defined as a radical and extreme surgical solution for the management of some locally advanced or locally recurrent malignant tumours of the pelvis. Untreated, such tumours have a consistently poor outcome with a median survival measured in months, which is often complicated by unremitting pelvic pain refractory to high‐dose opiates due to an enlarging tumour infiltrating pelvic muscles, nerves and bones [1]. Although first described as a palliative procedure, PE is now recognised as the only curative treatment for tumours refractory to chemotherapy, targeted therapies and radiotherapy, and has become the standard of care for very carefully selected patients [2].

The key goal of PE surgery is the *en bloc* resection of the tumour with all contiguously involved anatomical structures. This aims to achieve an oncologically complete surgical resection with negative cancer margins (R0‐resection), representing the most important indicator of survival and long‐term outcomes [3]. In more recent times, advances in surgical technique and multidisciplinary approaches have increasingly allowed more radical multi‐visceral resections to be safely performed, thereby expanding the boundaries of resectability in PE and providing a potential cure for patients previously considered inoperable [4].

There has also been a recognition that PE is an umbrella term, encompassing a wide variety of bespoke procedures, determined by the radiological boundaries of the tumour. Consequently, to create a common language among the PE community and establish more precise descriptions of the magnitudes of resection and therefore the extent of radicality, the UK Pelvic Exenteration Network (UKPEN) has established a PE lexicon, where cases are subdivided into either conventional or high‐complexity resections [5]. Conventional PE has been defined as the major surgical removal of all or most of the pelvic organs, while high‐complexity PE encompasses conventional exenteration with the addition of pelvic sidewall structures, such as major blood vessels and sciatic nerves, and/or bony structures, such as the sacrum and pubic bones. Nevertheless, even within the high‐complexity group, there may be some cases requiring a greater number of operative components to complete the resection and reconstruction stages—in a study validating the UKPEN PE lexicon, this implied the existence of a third ‘very’ high‐complexity group [2].

By its nature, rising complexity and a multiplicity of surgical components increase operating duration. This can escalate to a tumour being technically resectable, but due to the amount of theatre time required to achieve an R0 resection with optimal reconstruction, cases can potentially become inoperable if conducted over a single operating day. This may be due to logistical limitations, such as the impracticalities and quality/safety concerns of surgical specialists and clinical oncologists working through the night, or due to physiological constraints, resulting from substantial insensible fluid/blood loss and a desire to avoid prone operating at night. To address this, high‐complexity PE can be staged over more than the standard single operating day and encompass a second day. Patients undergo a temporary closure and remain intubated in intensive care overnight, allowing physiological stabilisation while facilitating rest and/or availability for surgical and clinical oncology teams—this is defined as high‐complexity two‐day/two‐stage PE.

Little is known about this approach, with data supporting its safety scarce. Operating time is known to be an independent risk factor for complications—particularly in procedures lasting longer than 10 h [6, 7]. In a systematic review, Cheng et al. (2018) observed that prolonged operating time was significantly associated with post‐operative complications, specifically in all pelvic surgery specialities [8]. Taken together, these findings suggest that longer elective surgery operating times may be associated with a worse post‐operative complication profile.

High‐complexity PE is usually conducted in a single operating day; however, since 2015, the Southampton Complex Cancer and Exenteration Team (SCCET) has undertaken high‐complexity two‐day/two‐stage PE operations for the most radical resections rather than recommending palliation. The present case‐control cohort study aims to evaluate the feasibility, safety and medium‐term outcomes of such procedures and compare these to lengthy single‐stage PE operations for less extensive disease but where there are similar logistical and physiological challenges.

## METHOD

### Patient cohorts

The UKPEN PE lexicon was applied to the prospectively maintained consecutive SCCET database (2010–2025) as previously described [2], to classify cases into high‐complexity or conventional PE, see File S1. All patients who had elective, planned, high‐complexity, two‐day/two‐stage PE were identified, included in this study, and their data retrieved. A matched‐pair control group of patients having PE but conducted over one operating day lasting ≥15 h were also included; cases lasting <15 h was excluded, with no other selection or matching criteria applied for this study. The experimental and control cohorts were further stratified into two a priori planned groups: patients who underwent single‐day or two‐day/two‐stage high‐complexity PE for colorectal tumours and those undergoing surgery for non‐colorectal malignancies. Any patients undergoing abdominal exenteration or palliative‐intent PE were excluded; however, patients on palliative pathways prior to their curative SCCET PE were included.

### Technique

Patients undergoing conventional and high‐complexity PE at SCCET have been previously described [2]. At our institution, all patients for a potential two‐day/two‐stage high‐complexity PE are selected pre‐operatively based on the number of ‘components’ within the planned operation, the extent of resection needed, as well as the reconstruction approaches planned as part of a multidisciplinary team (MDT). This decision is always made pre‐operatively based on experiential learning from previous prolonged one‐day cases—Table S1 provides a detailed description of case selection.

Although these discussions took place in conventional sub‐specialty cancer MDTs from 2010 to 2019, from 2019 onwards they were conducted in a specific SCCET Complex Cancer MDT for the delineation of suitability for radical surgery, the determination of the predicted operative roadmap and whether surgical adjuncts such as Intraoperative Electron Beam Radiotherapy (IOERT) and/or Heated Intraperitoneal Chemotherapy (HIPEC) would be required [9]. Patients requiring a two‐day/two‐stage PE are then planned over two consecutive days with typically a resection of the tumour on the first operating day, and reconstructions on the second day, with these lists already falling under the colorectal footprint. Upon completion of day one, temporary fascial and skin closure of the abdominal wound is affected, along with temporary perineal closure. Patients are then transferred from the operating theatre to the intensive care unit and cared for by a dedicated bedside nurse. Overnight they are kept intubated and ventilated, targeting a moderate‐to‐deep level of sedation. An inflammatory response is anticipated and blood pressure is maintained using noradrenaline. Intravenous crystalloid fluid resuscitation is kept to a minimum to reduce bowel oedema. Where there is concern about cardiovascular filling status, bedside focused echocardiography or minimally invasive cardiac output monitoring is utilised. Patients then return to theatre the following morning for completion of all components needed. All two‐day/two‐stage cases would have the cancer resection and some elements of reconstruction on day‐one, with the use of adjuncts like IOERT and complex composite soft tissue reconstructions with more than one tissue reconstruction method on day‐two. In all cases, we have opted to construct the ileal conduit on day‐one to avoid difficulties with anastomosing more oedematous bowel and ureters on day‐two, although final maturing of stomas including the urinary conduit may be conducted on day‐two. Transfer to intensive care was not following pelvic packing or for haemodynamic stabilisation; in all cases, it was deliberately planned to allow the completion of the required operative components.

### Outcome measures

The primary outcome was index admission mortality. Secondary outcomes were 30‐ and 90‐day mortality; overall survival; disease‐free survival; R0‐resection rate; index admission and overall major morbidity (Clavien‐Dindo complications ≥3a); index admission theatre utilisation; packed red cell units transfused; and, from 2021, consecutive longitudinal quality‐of‐life data collected using the generic validated EQ5D‐5L and decision regret scale patient‐reported outcome measures (PROMs) scored with relevant manuals [10, 11, 12]. More detailed definitions of outcomes are provided in File S1.

### Statistical analyses

No sample size calculation was conducted; this was dictated by the number of eligible patients within the database. Statistical analyses were performed using the Shapiro‐Wilk normality test with Mann‐Whitney U, Student's T‐test, Fisher's or Chi‐squared tests for continuous and categorical data, respectively. Kaplan‐Meier and log‐rank were used for time‐to‐event analysis with median follow‐up reported, including a survival sub‐analysis of colorectal cancer patients only. If loss to follow‐up occurred, patients were censored from time‐to‐event analysis at the date of their last known contact. Missing data were assumed to be missing completely at random, with a simple pairwise deletion strategy used throughout. No sensitivity analysis was performed. POSIT PBC RStudio (Version 2024.09.0 + 375) was used for all analysis.

This study corresponds to an early IDEAL stage 2B study by exploring a novel refinement of high‐complexity PE surgery, and the STROBE checklist was used for reporting—see File S2 [13, 14].

## RESULTS

### Patients and baseline characteristics

There were 373 cases in the SCCET database, of whom 255 underwent high‐complexity PE. Of these, 38 patients underwent one‐day PE ≥15 h, and 27 had two‐day/two‐stage high‐complexity PE. No cases were abandoned; a breakdown of components is given in Table S1. Both groups were highly comparable; however, there was a higher proportion of anal malignancies and locally recurrent cases in the two‐day/two‐stage high‐complexity PE group (*p* = 0.01 and *p* = 0.08, respectively). All two‐day/two‐stage high complexity PE cases were infralevator compared to 37% (14/38) in the one‐day PE group (*p* < 0.001); 89% (24/27) vs. 42% (16/38) had resection of bone beyond the coccyx only (p < 0.001). This was in constant with the two‐day/two‐stage patients undergoing more radical surgery; however, none of these had oncovascular resection/reconstruction of non‐expendable pelvic sidewall vessels, compared to 21% (8/38) in the one‐day group (*p* = 0.017). All cases were performed open with no minimally invasive techniques employed. No statistically significant differences were found between the groups in terms of other characteristics, including ASA grade, comorbidities, neoadjuvant treatments, use of IOERT or HIPEC. Further baseline demographics and clinical characteristics are summarised in Table 1.

**TABLE 1:** Baseline characteristics of the two groups.

Baseline patient characteristics	One‐day PE	Two‐day/two‐stage PE	*p*‐value
Characteristic			
Cohort sample sizes, *n* (% of total cohort)	38 (58)	27 (42)	
Sex, *n* (%)			
Female	14 (37)	7 (26)	0.43
Male	24 (63)	20 (74)	
Median age, years (IQR)	63 (13)	62 (15)	0.51
BMI, (IQR)	26.6 (6.6)	27.9 (9.0)	0.87
Smoking status, *n* (%)			
Current smoker	1 (3)	2 (7)	0.55
Ex‐smoker	12 (32)	10 (37)	
Non‐smoker	25 (66)	15 (56)	
Comorbidities, *n* (%)			
COPD/Asthma	4 (11)	4 (15)	0.71
Diabetes	2 (5)	3 (11)	0.64
Heart disease	2 (5)	1 (4)	1.00
Chronic kidney disease	5 (13)	0 (0)	0.07
Hypertension	11 (29)	6 (22)	0.58
ASA Grade, *n* (%)			
I	1 (3)	0 (0)	0.20
II	12 (32)	15 (56)	
III	24 (63)	12 (44)	
IV	1 (3)	0 (0)	
Diagnosis, *n* (%)			
Colorectal	36 (95)	20 (74)	0.01
Urological	1 (3)	0 (0)	
Gynaecological	0 (0)	2 (7)	
Anal	1 (3)	5 (19)	
Cancer status, *n* (%)			
Primary disease	20 (53)	8 (30)	0.08
Recurrent disease	18 (47)	19 (70)	
Oligometastatic disease	9 (24)	2 (7)	0.10
Neoadjuvant treatment	28 (73)	18 (67)	0.59
Initially on palliative pathway^a^	6 (16)	6 (22)	0.53
Type of pelvic exenteration, *n* (%)			
Supralevator	14 (37)	0 (0)	<0.001
Infralevator	24 (63)	27 (100)	
IOERT	21 (55)	15 (56)	1.00
HIPEC	3 (1)	0 (0)	1.00
Bone resection	16 (42)	24 (89)	<0.001
Non‐expendable vessel resection/reconstruction^b^	8 (21)	0 (0)	0.017

Abbreviations: ASA, American Society of Anaesthesiologists; BMI, body mass index; COPD, chronic obstructive pulmonary disease; HIPEC, hyperthermic intraperitoneal chemotherapy; IQR, interquartile range; IOERT, intraoperative electron radiotherapy.

^a^ Patients judged to be palliative prior to evaluation by the Southampton Complex Cancer and Exenteration Team.

^b^ Non‐expendable vessels are defined as the aorta, inferior vena cava, common or external iliac arteries and/or veins.

### Survival

**FIGURE 1:**
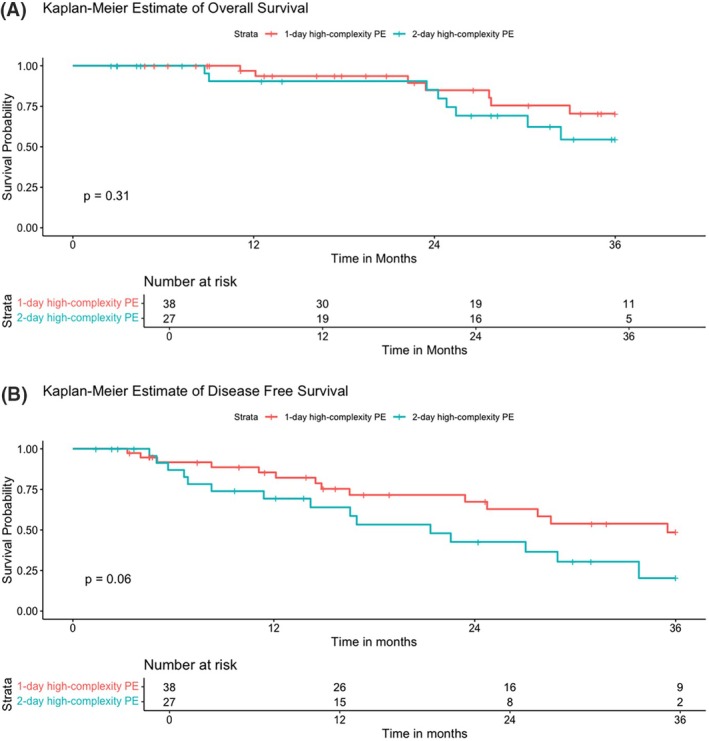
(A) Kaplan‐Meier, log rank and life‐table for overall survival, red line denoting one‐day pelvic exenteration (PE), and blue line denoting two‐day/two‐stage PE; (B) disease‐free survival.

There were no 90‐day mortalities in either group; 3‐year overall survival was 54.4% for two‐day/two‐stage PE and 70.5% for one‐day PE (*p* = 0.31); disease‐free survival was 20.3% and 48.5% (*p* = 0.06), respectively. Kaplan‐Meier analysis is shown in Figure 1. Figure S1 demonstrates a sub‐analysis of colorectal cancer patients only, demonstrating both overall survival and disease‐free survival were more similar between the groups, *p* = 0.73 and *p* = 0.18, respectively.

### Margin status and complications

R0‐resection margins were achieved in 82% (22/27) of the two‐day/two‐stage PE group and 76% (29/38) in the one‐day group, with no significant difference (*p* = 0.76). Major complications during the index admission and overall major complications until last follow‐up were not significantly different: index admission was 52% (14/27) and 42% (16/38), *p* = 0.46; and overall complications were 56% (15/27) to 47% (18/38), *p* = 0.62, respectively.

### Resource use

There were significant differences in hospital resource use with two‐day/two‐stage PE having longer median operating times (1390 vs. 988 min, *p* < 0.001); critical care lengths of stay (8 vs. 5 days, *p* = 0.002); and overall lengths of stay (37 vs. 27 days, *p* = 0.07). Mean units of red cells transfused during admissions were significantly higher in the one‐day group (7.29 vs. 5.78, *p* < 0.001). Both groups had a median follow‐up of at least 23 months (*p* = 0.42). Further details on outcomes are shown in Table 2.

**TABLE 2:** Outcomes table.

Outcomes	One‐day PE	Two‐day/two‐stage PE	*p*‐value
Sample size, *n* (%)	38 (58)	27 (42)	
Survival, *n* (%)			
90‐day mortality	0 (0)	0 (0)	1.00
3‐year OS	70.5	54.4	0.31
3‐year DFS	48.5	20.3	0.06
Margin status^a^, *n* (%)			
R0	29 (76)	22 (82)	0.76
R1	9 (24)	5 (19)	
Continuous R1	4* (44)	4 (80)	0.30
Discontinuous R1	5* (56)	1 (20)	
Primary tumour R1	4 (11)	0 (0)	0.13
Recurrent tumour R1	5 (13)	5 (19)	0.72
Morbidity, *n* (%)			
Index admission major complications	16 (42)	14 (52)	0.46
Overall major complications	18 (47)	15 (56)	0.62
Resource use			
Median length of surgery, minutes (IQR)	988 (216)	1390 (201)	<0.001
Mean units of packed red cells used during admission, units (SD)	7.29 (7.48)	5.78 (3.98)	<0.001
Median intensive care LOS, days (IQR)	3 (5.5)	5 (4)	0.08
Median high‐dependency LOS, days (IQR)	3 (2)	3 (5.5)	0.36
Median total critical care LOS, days (IQR)	5 (4)	8 (4)	0.002
Median overall LOS, days (IQR)	27 (18)	37 (20.5)	0.07
Median follow up time, months (IQR)	22.8 (29.6)	24.2 (21.9)	0.42

Abbreviations: DFS, disease‐free survival; IQR, interquartile range; LOS, length of stay; OS, overall survival; PE, pelvic exenteration; SD, standard deviation.

### Patient‐reported outcome measures

**FIGURE 2:**
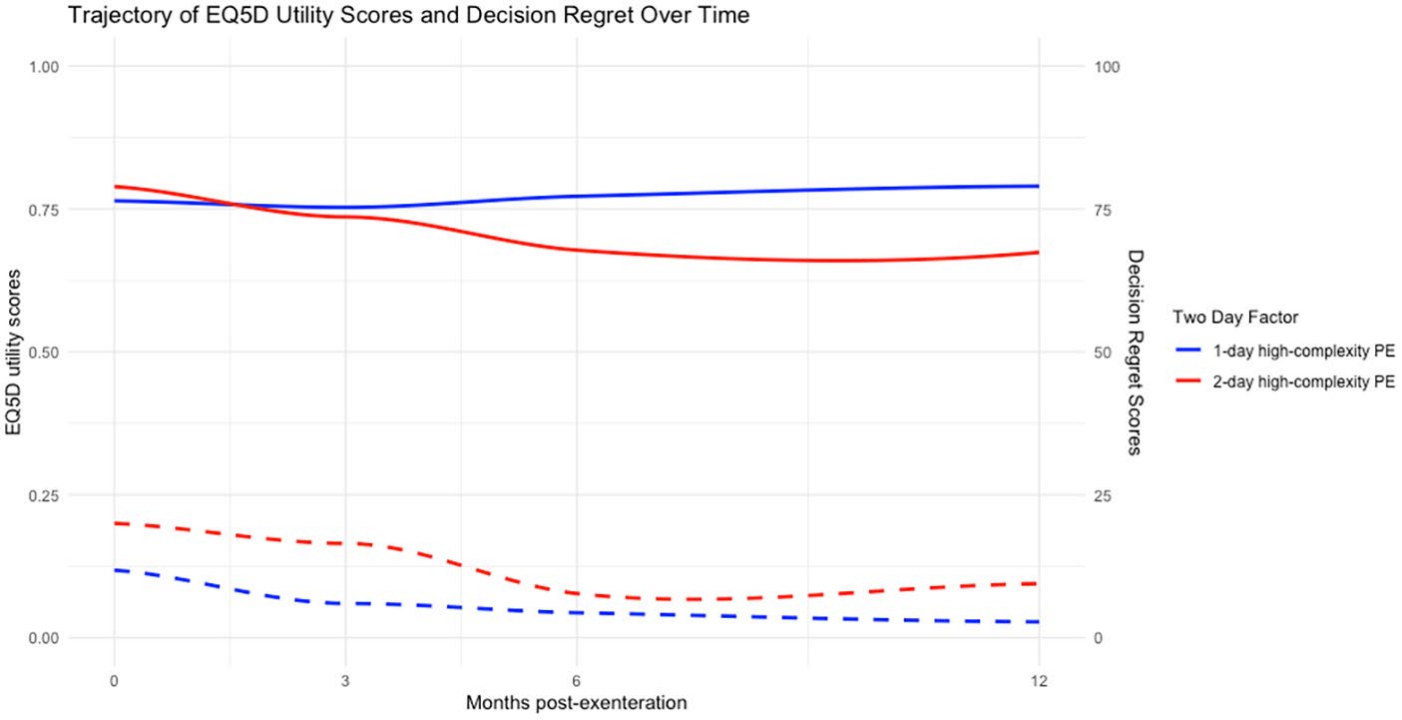
Locally estimated scatterplot smoothing regression lines of longitudinal EQ5D‐5L utility scores (non‐dashed line) and decision regret scale scores (dashed line), for one‐day PE in blue and two‐day/two‐stage PE in red. The left y‐axis gives EQ5D‐5L utility scores with 1.0 representing maximum utility, while the right axis gives decision regret scale scores with 100 representing maximal decision regret.

PROM data were available for 40.7% (11/27) of two‐day/two‐stage PE cases and 28.9% (11/38) of one‐day PE cases. Longitudinal EQ5D‐5L utility and decision regret scale scores are plotted in Figure 2. No statistically significant differences were seen at any time point using individual scores from PROMs. Full details are given in Table S2.

## DISCUSSION AND CONCLUSIONS

The acceptance of PE surgery and its more radical subsets of high‐complexity PE has been slow but steady in the medical community. Driven in part by the bleak outcomes of available non‐surgical alternatives and the excellent outcomes reported from specialist international units, it is increasingly acknowledged that these multi‐visceral operative interventions, while radical and life‐changing, represent the standard of care in very carefully selected patients. However, as the complexity of resections needed to achieve a negative resection margin, and the intricacy of reconstructions subsequently required have grown, so has the duration of surgery, necessitating procedures to be staged over more than the conventional single operating day. In the current study, we have evaluated the safety, as well as short and medium‐term outcomes in patients having two‐day/two‐stage high‐complexity PE vs. one‐day high‐complexity PE.

Despite the magnitude of these operations, there were no 90‐day mortalities, implying that two‐day/two‐stage PE is safe, falling below the ≤6% international benchmark defined by Brown et al. 2024, and indeed their ≤53% index admission major complication rate [15]. Benchmarks for overall lengths of stay, critical care admissions and duration of surgery were exceeded; however, Brown et al. deliberately excluded high‐risk cases from their benchmarking criteria, while the present study captures the most challenging patients SCCET has managed, who undeniably have required additional health resources. Despite this complexity, the R0‐resection rates reported are close to Brown's ≥ 77% benchmark, and of note, over half the patients in the current study had IOERT. This modality is known to mitigate against R1 resections by potentially achieving an oncological outcome equivalent to R0 resections [16, 17]. This enables more borderline cases to become eligible for curative treatment, and indeed 18% of cases in this study were initially considered to have been palliative by referring institutions due to the likelihood of an R1/R2 resection.

Although not significant (*p* = 0.76), the R0‐resection rates were counterintuitively 6% better in the two‐day/two‐stage PE group, who had more complex disease requiring additional operative components. This may be due to more of these cases being performed once SCCET had become established and more experienced as a PE unit, but also could represent a human factor element. PE surgery is known to be physically demanding for surgeons, with heart rates increasing up to 106% from baseline during dissection [18]. In other high‐stress environments, surgical teams can become ineffective without rest [19]. In the two‐day/two‐stage cases, delivering the specimen becomes the critical target for the first stage. This avoids a partially denuded tumour, which can cause physiological issues in intensive care, and also mitigates against surgeon timing and logistical anxieties when additional teams are required for reconstruction or IOERT delivery. Thus, more focus on the resection margin is facilitated compared to a prolonged one‐day/one‐phase approach. In addition, complex reconstruction techniques are avoided in the early hours when reconstructive teams and surgeons may be more sleep deprived, a variable known to be associated with impaired technical skill performance, higher complication rates and increased mortality in the general surgical literature [20].

Despite the slightly better R0‐resection rates in the two‐day/two‐stage group, the 3‐year disease‐free survival was borderline significantly inferior (*p* = 0.06). Significantly more anal cancers (*p* = 0.01) and recurrent cases occurred in the two‐day/two‐stage cohort (*p* = 0.08). These disease groups are known to have more aggressive tumour biology necessitating more radical resections and consequently more extensive reconstructions; it is therefore not surprising that such cases more frequently require the two‐day/two‐stage approach. They are also known to have oncologically inferior outcomes compared to primary PE for locally advanced rectal cancers [3, 21], with more encouraging comparisons of survival when only colorectal cancer patients were included, albeit with still more recurrent cases in the two‐day/two‐stage group. Alternatively, the increase in operative components and higher radicality of surgery may induce a longer‐lasting and more intensive systemic inflammatory response, which potentially could influence distant tumour proliferation—already recognised in colorectal cancer surgery [22].

Patients received on average 1.51 units of blood more in the one‐day group (*p* < 0.001); this is likely explained by the higher numbers of non‐expendable pelvic vessel resections/reconstructions (e.g. common and external iliac vessels, as opposed to expendable internal iliac vessels) and has been reported elsewhere in the PE literature [23]. In our experience, oncovascular PE cases are usually supralevator and do not add as much time as infralevator operations. The median intensive care stay for the two‐day/two‐stage group was 2 days longer, approaching significance (*p* = 0.08). This suggests that these patients may be intubated for more time, but despite this anaesthetic and respiratory complications were not found to be issues in this study. Such events occurred in only 3% of SCCET PE patients overall, with empty pelvis syndrome and urological complications being more prevalent [24].

In the field of PE, there is a rising consensus that health‐related quality of life (HrQoL) outcomes should be viewed as importantly as oncological survival [4]. Currently, there are no PROMs with content validity to evaluate HrQoL for all patients with different malignancies undergoing PE [25]. The generic PROMs used in the present study as a substitute are therefore less sensitive at capturing differences, with no significant variations in HrQoL at any time point seen. It is possible, however, to comment on HrQoL trends, and there appears to be either an absent or slower recovery to baseline HrQoL in the two‐day/two‐stage PE group. This is not unexpected, as patients undergoing less radical resections, for example, conventional PE, can have significantly better pain and mobility scores post‐operatively [2], however, reassuringly, decisional regret was low in both groups. This is despite the high levels of major morbidity and prolonged hospital admissions and may be explained by the phenomenon that for patients undergoing PE, it is a matter of survival, enabling them to tolerate reduced HrQoL, as they are grateful to be alive [26]. When considered against the alternative of no surgery, which is known to have poor survival with declining HrQoL until death, this data is particularly encouraging [27].

This study has limitations; it has a relatively small sample size based on a single tertiary academic referral unit, which is unlikely to be representative of hospitals conducting conventional PE only. Minor morbidity was not analysed as it was very common in both groups; therefore, there was a focus on major complications. The comparison is flawed due to confounding, as patients selected for the two‐day/two‐stage PE are, by their nature, undergoing surgery of a higher magnitude than the one‐day cohort. However, it is encouraging that, barring resource use, no outcomes of interest were significantly different. Theatre utilisation is clearly increased per case, with the impact on wider patient flow and waiting lists not evaluated. This has been justified as a centre delivering complex surgical oncology, with both cohorts in this study included in a cost‐utility analysis demonstrating that high‐complexity PE overall was well within UK commissioning willingness‐to‐pay thresholds compared to palliative care. This was principally due to the younger age of patients undergoing PE surgery and the dismal survival and PROMs of individuals being palliated with locally advanced pelvic cancer [2]. Finally, to the best of our knowledge, after performing a systematic literature review, this is the first study to report on the outcomes of two‐day/two‐stage PE.

Two‐day/two‐stage PE was found to be safe and feasible and may represent the only curative option for highly selected patients with intractable pelvic cancer. Greater morbidity and resource use were noted in these patients compared to one‐day ≥15 h high‐complexity PE. Although equivalent R0 resections were obtained, medium‐term oncological outcomes appeared poorer in patients having two‐day/two‐stage interventions, a finding potentially explainable by the increased frequency of locally recurrent anal cancers in the two‐day/two‐stage cohort—a disease more likely to require more extensive surgery.

The SCCET decision‐making process for two‐day/two‐stage PE is currently based on MDT experience and consensus; further refinement of patient selection, surgical sequencing and resource allocation should improve upon the outcomes of two‐day/two‐stage PE reported here. Future collaborative studies guided by a standardised core data set, enabling more consistent data collection, will chart the way forward [28].

## AUTHOR CONTRIBUTIONS

**Charles T. West:** Data curation; formal analysis; investigation; project administration; software; visualization; writing – original draft; writing – review and editing. **Yousif Salem:** Investigation; writing – original draft. **Siddharth Jain:** Writing – review and editing; investigation. **Lewis Matthews:** Writing – review and editing. **Julian Smith:** Conceptualization; resources. **Marios Nicolaou:** Conceptualization; resources. **Hideaki Yano:** Conceptualization; investigation; supervision; resources; writing – review and editing. **Malcolm A. West:** Conceptualization; investigation; validation; supervision; resources; writing – review and editing. **Alex H. Mirnezami:** Conceptualization; methodology; supervision; resources; writing – review and editing.

## FUNDING INFORMATION

The data for this study were collected as part of a PhD thesis of the first author, which was jointly funded by Bowel Research UK, PLANETS Cancer Charity and Penguins Against Cancer.

## CONFLICT OF INTEREST STATEMENT

The authors declare no conflicts of interest.

## ETHICS STATEMENT

Ethical approval was granted by NHS North East—Newcastle and North 2 Research Ethics Committee (REC: 22/NE/0032).

## PATIENT CONSENT

Patients recruited for quality‐of‐life questionnaires completed informed consent forms, and permission to analyse data for patients in the database prior to 2021 was granted through the above ethics.

## Supporting information


Figure S1.

File S1.

File S2.

Table S1:

Table S2:

## Data Availability

The data that support the findings of this study are available on request from the corresponding author. The data are not publicly available due to privacy or ethical restrictions.
