# Supplementary material for: Prospective case‐control cohort analysis of two‐day/two‐stage pelvic exenteration surgery: Safety, feasibility, acceptability and medium‐term outcomes
Source: Colorectal Dis. 2025 Dec 29;28(1):e70353. doi: 10.1111/codi.70353 (PMC12748039; doi:10.1111/codi.70353)
Supplement: Supplementary file 1 — Figure S1. [file CODI-28-0-s003.docx]

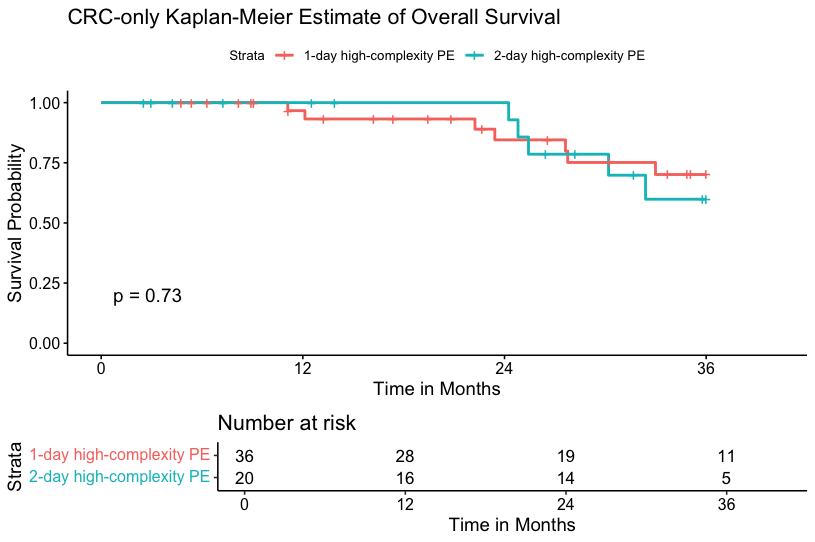

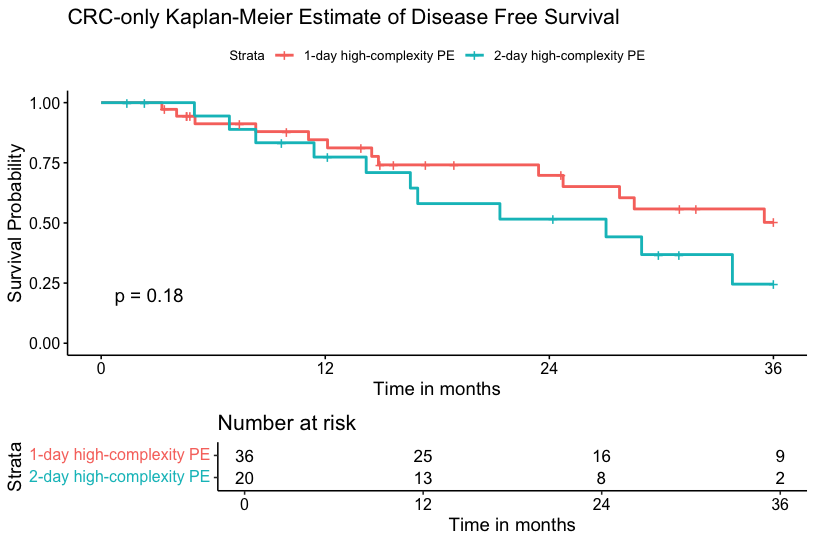


B

A

Figure S1 – Stratified survival sub-analysis of only colorectal cancer patients. A) Kaplan-Meier, log rank and life-table for overall survival, red line denoting one-day pelvic exenteration (PE), and blue line denoting two-day/two-stage PE; B) Disease-free survival.
