# Supplementary material for: Prospective case‐control cohort analysis of two‐day/two‐stage pelvic exenteration surgery: Safety, feasibility, acceptability and medium‐term outcomes
Source: Colorectal Dis. 2025 Dec 29;28(1):e70353. doi: 10.1111/codi.70353 (PMC12748039; doi:10.1111/codi.70353)
Supplement: Supplementary file 2 — File S1. [file CODI-28-0-s004.docx]

**Supplementary File 1 – Definitions:**

*Descriptors:*

- Pelvic exenteration (PE) – any resection involving two or more organs or compartments within the greater or lesser pelvis
- Complexity was defined as per the UK Pelvic Exenteration Network lexicon
  - High-complexity PE: ≥P2, ≥A4, ≥SV1, ≥SN1, ≥PM2, E1, E2, E3, and E4. Coccygectomy alone and E5 resections, including the concurrent removal of inguinal or hepatic oligometastic disease, appendicectomy, and resection of involved small bowel or colon pulled into a tumour mass, were not considered when defining pelvic resection complexity.
  - Conventional PE: ≤P1, ≤A3, ≤C3, ≤SV0, ≤SN0, ≤PM1

*Outcomes:*

- Overall survival – open-ended time between surgery and death, utilising NHS Summary Care Records for UK mainland patients, with referring non-NHS institutions contacted to confirm survival if not held by Southampton.
- Disease-free survival – Kaplan-Meier open-ended time until any cancer recurrence appeared.
- R0-resection – at least 1mm margin from any malignant cells, be they continuous with the main tumour, or viable discontinuous tumour cells, as per contemporary [UK Royal College of Pathology Guidelines](https://www.rcpath.org/static/c8b61ba0-ae3f-43f1-85ffd3ab9f17cfe6/c19a5cd7-3485-44c2-b5e1c87154830582/G049-Dataset-for-histopathological-reporting-of-colorectal-cancer.pdf) for colorectal cancer, but adapted to other tumour types. Note that pelvic exenteration and hyperthermic intraperitoneal chemotherapy for disseminated peritoneal disease were excluded from this analysis.
  - Continuous R1-resection – where the R1 margin is continuous with the main tumour.
  - Discontinuous R1-resection – where the R1 margin is not continuous with the main tumour, constituting tumour fragmentation, tumour deposits, or malignant encapsulated or non-encapsulated lymph nodes.
- Index admission major morbidity – a Clavien-Dindo complication ≥3a during the admission immediately following pelvic exenteration
- Overall major morbidity – index admission major morbidity with the addition of any Clavien-Dindo complication ≥3a attributable to pelvic exenteration occurring following discharge, until the last date of follow-up or death. This did not include complications arising due to disease recurrence.
