# Supplementary material for: Prospective case‐control cohort analysis of two‐day/two‐stage pelvic exenteration surgery: Safety, feasibility, acceptability and medium‐term outcomes
Source: Colorectal Dis. 2025 Dec 29;28(1):e70353. doi: 10.1111/codi.70353 (PMC12748039; doi:10.1111/codi.70353)
Supplement: Supplementary file 3 — File S2. [file CODI-28-0-s005.docx]

**Supplementary File 2 – STROBE checklist:**

|  | Item | Recommendation | Location in subheading |
| --- | --- | --- | --- |
| **Title and abstract** | 1 | (*a*) Indicate the study’s design with a commonly used term in the title or the abstract | Abstract |
|  |  | (*b*) Provide in the abstract an informative and balanced summary of what was done and what was found | Abstract |
| Introduction | | |  |
| Background/rationale | 2 | Explain the scientific background and rationale for the investigation being reported | Introduction as a whole |
| Objectives | 3 | State specific objectives, including any prespecified hypotheses | Introduction paragraph 6 |
| Materials and Methods | | |  |
| Study design | 4 | Present key elements of study design early in the paper | Method paragraph 1 |
| Setting | 5 | Describe the setting, locations, and relevant dates, including periods of recruitment, exposure, follow-up, and data collection | Method paragraph 1 and Supplementary File 1 |
| Participants | 6 | (a) *Case-control study*—Give the eligibility criteria, and the sources and methods of case ascertainment and control selection. Give the rationale for the choice of cases and controls | Method paragraph 1 |
|  |  | (b) *Case-control study*—For matched studies, give matching criteria and the number of controls per case | Method paragraph 1 |
| Variables | 7 | Clearly define all outcomes, exposures, predictors, potential confounders, and effect modifiers. Give diagnostic criteria, if applicable | Method paragraph 3 and Supplementary File 1 |
| Data sources/ measurement | 8* | For each variable of interest, give sources of data and details of methods of assessment (measurement). Describe comparability of assessment methods if there is more than one group | Method paragraphs 1, 2, and 3, and Supplementary File 1 |
| Bias | 9 | Describe any efforts to address potential sources of bias | Supplementary File 1, Method paragraph 4 |
| Study size | 10 | Explain how the study size was arrived at | Method paragraph 4 |
| Quantitative variables | 11 | Explain how quantitative variables were handled in the analyses. If applicable, describe which groupings were chosen and why | Method paragraphs 1, 4, and Supplementary File 1 |
| Statistical methods | 12 | (*a*) Describe all statistical methods, including those used to control for confounding | Method paragraph 4 |
|  |  | (*b*) Describe any methods used to examine subgroups and interactions | Method paragraphs 1 and 4 |
|  |  | (*c*) Explain how missing data were addressed | Method paragraph 4 and Supplementary File 1 |
|  |  | (d) *Case-control study*—If applicable, explain how matching of cases and controls was addressed | Method paragraph 1 |
|  |  | (*e*) Describe any sensitivity analyses | Method paragraph 4 |
| **Results** | | | |
| Participants | 13* | (a) Report numbers of individuals at each stage of study—eg numbers potentially eligible, examined for eligibility, confirmed eligible, included in the study, completing follow-up, and analysed | Results paragraph 1 |
|  |  | (b) Give reasons for non-participation at each stage | Results paragraph 1 |
|  |  | (c) Consider use of a flow diagram | Not included |
| Descriptive data | 14* | (a) Give characteristics of study participants (eg demographic, clinical, social) and information on exposures and potential confounders | Table 1 and results paragraph 1 |
|  |  | (b) Indicate number of participants with missing data for each variable of interest | Table 1 and Table S2 |
| Outcome data | 15* | *Case-control study*—Report numbers in each exposure category, or summary measures of exposure | Table 1 and Results paragraph 1 |
| Main results | 16 | (*a*) Give unadjusted estimates and, if applicable, confounder-adjusted estimates and their precision (eg, 95% confidence interval). Make clear which confounders were adjusted for and why they were included | Throughout results |
|  |  | (*b*) Report category boundaries when continuous variables were categorized | N/A |
|  |  | (*c*) If relevant, consider translating estimates of relative risk into absolute risk for a meaningful time period | N/A |
| Other analyses | 17 | Report other analyses done—eg analyses of subgroups and interactions, and sensitivity analyses | Throughout results |
| **Discussion** | | | |
| Key results | 18 | Summarise key results with reference to study objectives | Discussion paragraph 1 |
| Limitations | 19 | Discuss limitations of the study, taking into account sources of potential bias or imprecision. Discuss both direction and magnitude of any potential bias | Discussion paragraph 7 |
| Interpretation | 20 | Give a cautious overall interpretation of results considering objectives, limitations, multiplicity of analyses, results from similar studies, and other relevant evidence | Throughout discussion and concusions |
| Generalisability | 21 | Discuss the generalisability (external validity) of the study results | Discussion paragraph 7 |
| **Other information** | | | |
| Funding | 22 | Give the source of funding and the role of the funders for the present study and, if applicable, for the original study on which the present article is based | Funding section in title page |

**Note:** Adapted from [www.strobe-statement.org](https://protect.checkpoint.com/v2/___http://www.strobe-statement.org___.bXQtcHJvZC1jcC1ldXcyLTE6dW5pdmVyc2l0eWhvc3BpdGFsc291dGhhbXB0b246YzpvOjUwZDA4YTg1MWY1OTI0OWIxNTlmMGQ0Mjk4ZGFiZTZjOjY6NGUwZToxYTYxYWZkY2E3ZTc0Yzk5NDYxYzlhYTJlYTlhZjdiYjViNGQ5YjdhMDI5MDVhMTE0N2JhZDExOWU2ZmQzZWI2OnA6VDpO) with references to cohort and cross-sectional studies removed.
