# Supplementary material for: Prospective case‐control cohort analysis of two‐day/two‐stage pelvic exenteration surgery: Safety, feasibility, acceptability and medium‐term outcomes
Source: Colorectal Dis. 2025 Dec 29;28(1):e70353. doi: 10.1111/codi.70353 (PMC12748039; doi:10.1111/codi.70353)
Supplement: Supplementary file 4 — Table S1: [file CODI-28-0-s002.docx]

| **Case number** | **Indication** | **Previous resections** | **Lexicon coding** | **Day 1 steps completed (minutes)** | **Day 2 steps**  **Completed (minutes)** |
| --- | --- | --- | --- | --- | --- |
| 1 | LRRC | Total supralevator PE | A4 C2 ltSV1 rtSV1 ltSN1 PM1 + two small bowel loops | Adhesiolysis, all resections, enteric anastomoses, and omentoplasty (621) | Gracilis flap, and biological mesh (357) |
| 2 | LRRC | Local rectal resection | P3 A3 C2 ltSV1 rtSV1 PM1 + appendicectomy | Adhesiolysis, all resections, omentoplasty, Wallace plate (893) | IOERT, VRAM, abdominal wall biological mesh, conduit matured (539) |
| 3 | LRRC | Anterior resection, completion colectomy | P3 A3 C2 ltSV3 rtSV3 PM1 | Adhesiolysis, abdominal resection, Wallace conduit (917) | Perineal sacrectomy, biological mesh reconstruction, gluteal flap (332) |
| 4 | Recurrent anal SCC | Hysterectomy, oophorectomy, left nephrectomy, cystectomy | A3 C3 ltSV3 PM1 + small bowel resection | Adhesiolysis, all resections, enteric anastomosis (817) | IOERT, VY and gracilis flaps, biological mesh reconstruction (661) |
| 5 | LRRC | Anterior resection | A3 C2 ltSV3 rtSV3 PM1 | Adhesiolysis, all resections, haemostatic pelvic packing (890) | Reversal loop ileostomy, biological mesh reconstruction, Wallace conduit, omentoplasty (373) |
| 6 | LRRC | Anterior resection | P3 A3 C2 ltSV3 rt SV3 PM1 + appendicectomy | Adhesiolysis, all resections, Wallace conduit (891) | IOERT, VRAM, omentoplasty, biological mesh AWR (511) |
| 7 | LRRC | Abdominoperineal resection | A4 C2 ltSV3 rtSV1 PM1 + appendicectomy | Adhesiolysis, all resections, Wallace plate (975) | IOERT, biological mesh perineum, conduit matured (540) |
| 8 | LRRC | Prostatectomy, ELAPE with VRAM | A5 C2 ltSV1 rtSV1 PM1 + small bowel resection | Adhesiolysis, all resections, Wallace conduit, omentoplasty (809) | IOERT, biological mesh, PAP flap (530) |
| 9 | LRRC | Anterior resection, right hemi-hepatectomy | P3 A3 C2 ltSV2 rtSV1 ltSN3 PM1 + appendicectomy | Adhesiolysis, all resections, Wallace conduit, omentoplasty (885) | IOERT, VRAM, biological mesh AWR (538) |
| 10 | LRRC | ELAPE with biological mesh | P3 A3 C2 ltSV3 rtSV1 ltSN1 PM2 E2 + malignant perineal small bowel fistula + appendicectomy | Adhesiolysis, all resections, biological mesh, omentoplasty, Wallace conduit (1086) | PAP flap (363) |
| 11 | Recurrent anal SCC | ELAPE with bilateral gracilis flaps | P3 A3 C2 rtSV3 rtSN5 PM2 | Adhesiolysis, all resections, Wallace conduit, omentoplasty (944) | IOERT, biological mesh perineum, gluteal VY flap (436) |
| 12 | LARC | Nil | P3 A3 C2 ltSV1 rtSV3 PM2 E2 + appendicectomy | Adhesiolysis, all resections, omentoplasty, Wallace conduit (884) | IOERT, biological mesh perineum, gracilis and PAP flap (563) |
| 13 | LARC | Nil | P3 A3 C2 ltSV2 rt SV3 PM1 + appendicectomy | All resections, omentoplasty, Wallace conduit (780) | IOERT, VRAM, PAP, and gracilis flaps (390) |
| 14 | Recurrent anal SCC | ELAPE with VRAM | P3 A5 C2 ltSV1 rt SV2 rtSN3 PM2 (bilateral) + small bowel resection + previous VRAM resected | Adhesiolysis, all resections, Wallace conduit, omentoplasty (810) | IOERT, bilateral gracili and PAP flaps (460) |
| 15 | Recurrent cervical SCC | Trachelectomy, right nephrectomy | P4 A3 C2 ltSV1 rtSV3 rtSN5 PM2 + appendicectomy | Adhesiolysis, all resections, omentoplasty, Wallace conduit (925) | IOERT, bilateral VRAM, perineal biological mesh, left PAP and gracilis flaps (430) |
| 16 | LARC | Nil | P4 A3 C2 ltSV3 rtSV3 PM1 + appendicectomy | All resections, Wallace plate, omentoplasty (900) | VRAM + bilateral component separation, conduit matured (450) |
| 17 | Recurrent anal SCC | Nil | A4 C3 ltSV1 rtSV3 PM2 + appendicectomy | All resections, Wallace plate, omentoplasty (735) | IOERT, VRAM, conduit matured (495) |
| 18 | LARC | Nil | P3 A3 C2 ltSV1 rtSV3 PM2 + appendicectomy | All resections, Wallace plate, omentoplasty (930) | Parastomal hernia repair, conduit matured, perineal biological mesh, gluteal advancement flap (365) |
| 19 | LARC | Nil | P4 A3 C2 ltSV3 rtSV3 PM1 + appendicectomy | All resections, omentoplasty (960) | Wallace conduit, VRAM (505) |
| 20 | LRRC | ELAPE with biological mesh | P3 A3 C2 ltSV1 rtSV3 rtSN5 PM2 + appendicectomy | Adhesiolysis, all resections, omentoplasty, Wallace plate (992) | IOERT, VRAM, conduit matured, biological mesh AWR (549) |
| 21 | LRRC | Anterior resection | P4 A3 C2 ltSV3 rtSV3 ltSN2 rtSN2 PM2 (bilateral) + appendicectomy | Adhesiolysis, all resections, Wallace plate (1002) | IOERT, VRAM, omentoplasty, conduit matured (580) |
| 22 | LRRC | Total infralevator pelvic exenteration | P3 A3 C2 rtSV3 rtSN5 PM2 + small bowel resection | Adhesiolysis, all resections (770) | Enterotomy resection and anastomosis, VRAM, gracilis, and VY advancement flaps, biological mesh perineum (630) |
| 23 | Recurrent anal SCC | ELAPE with VRAM | P3 A3 C2 rtSV3 rt SN3 PM2 + previous VRAM resected | Adhesiolysis, all resections, omentoplasty, Wallace conduit (804) | IOERT, biological mesh perineum, gracilis flap (445) |
| 24 | LARC | Pan-proctocolectomy with pouch | P3 A3 C2 ltSV3 rtSV3 PM1 | Adhesiolysis, specimen removed, Wallace plate (994) | VRAM, and biological mesh perineum, conduit matured (560) |
| 25 | Recurrent vulval SCC | Anterior vulvectomy | A3 C3 rtSV2 PM1 + right groin lymphadenectomy + appendicectomy | All resections, Wallace plate fashioned (635) | VRAM, omentoplasty, ileal conduit matured, and end colostomy (452) |
| 26 | LARC | Nil | P3 A3 C2 rtSV1 PM1 E2 + appendicectomy | All resections, omentoplasty, Wallace plate fashioned, end colostomy (744) | VRAM, and ileal conduit matured (503) |
| 27 | LARC | Nil | P2+3 A3 C2 ltSV2 rtSV3 PM1 + appendicectomy + liver metastectomy + inguinal lymph node dissection | All abdominopelvic resections, Wallace plate fashioned (976) | VRAM, inguinal lymph node dissection, and ileal conduit matured (624) |

Table S1 – Breakdown of day-one and day-two components for two-day/two-stage pelvic exenteration (PE) cases. LRRC = locally recurrent rectal cancer, LARC = locally advanced rectal cancer, SCC = squamous cell carcinoma, ELAPE = extralevator abdominoperineal excision, VRAM = vertical rectus abdominis myocutaneous flap, IOERT = intra-operative electron radiotherapy, PAP = popliteal artery perforator flap, AWR = abdominal wall reconstruction. PE lexicon is as per Burns et al 2023 (5).
