# Supplementary material for: Prospective case‐control cohort analysis of two‐day/two‐stage pelvic exenteration surgery: Safety, feasibility, acceptability and medium‐term outcomes
Source: Colorectal Dis. 2025 Dec 29;28(1):e70353. doi: 10.1111/codi.70353 (PMC12748039; doi:10.1111/codi.70353)
Supplement: Supplementary file 5 — Table S2: [file CODI-28-0-s001.docx]

| PROM Score | Months post-PE *(n)* | | | | | | | |
| --- | --- | --- | --- | --- | --- | --- | --- | --- |
|  | Baseline – 0 | | 3 | | 6 | | 12 | |
| **Decision Regret Scale, medians (IQR), p-value** | | | | | | | | |
| two-day/two-stage PE | 15 (17.5) | 0.36 | 7.5  (31.3) | 0.31 | 5  (10) | 0.43 | 5  (8.75) | 0.15 |
| one-day PE | 15  (22.5) |  | 5  (10) |  | 0  (10) |  | 0  (5) |  |
| **EQ5D-5L, medians (IQR), p-value** | | | | | | | | |
| *5L-Utility score* | | | | | | | | |
| two-day/two-stage PE | 0.86  (0.16) | 0.79 | 0.7  (0.16) | 0.40 | 0.81  (0.18) | 0.97 | 0.79  (0.40) | 0.96 |
| one-day PE | 0.80  (0.27) |  | 0.72  (0.21) |  | 0.75  (0.13) |  | 0.81  (0.06) |  |
| *Visual analogue score* | | | | | | | | |
| two-day/two-stage PE | 75  (30) | 1.00 | 70  (19.5) | 0.89 | 70  (12.5) | 0.36 | 65  (22.5) | 0.72 |
| one-day PE | 70  (26.5) |  | 70  (25) |  | 77.5  (16.2) |  | 77.5  (25) |  |
| *Mobility* | | | | | | | | |
| two-day/two-stage PE | 1  (1) | 0.48 | 3  (1) | 0.48 | 2  (1.5) | 0.90 | 2  (2.5) | 0.93 |
| one-day PE | 2  (1) |  | 2.5  (1.75) |  | 3  (1.25) |  | 3  (1) |  |
| *Self-care* | | | | | | | | |
| two-day/two-stage PE | 1  (0) | 0.17 | 2  (1) | 0.20 | 1  (1) | 0.44 | 2  (1) | 0.28 |
| one-day PE | 1  (0) |  | 1  (0.75) |  | 1  (0.25) |  | 1  (1) |  |
| *Usual activities* | | | | | | | | |
| two-day/two-stage PE | 1  (1) | 0.82 | 3  (2) | 0.15 | 2  (1) | 0.67 | 2  (1.75) | 0.80 |
| one-day PE | 1  (1) |  | 2  (1) |  | 3  (1.5) |  | 2  (1) |  |
| *Pain / discomfort* | | | | | | | | |
| two-day/two-stage PE | 3  (1.5) | 0.68 | 2  (1) | 0.64 | 2  (0.5) | 0.45 | 2  (0.75) | 0.75 |
| one-day PE | 2  (1) |  | 2  (0.75) |  | 2  (1.25) |  | 2  (1) |  |
| *Anxiety / depression* | | | | | | | | |
| two-day/two-stage PE | 2  (0.5) | 0.94 | 1  (1) | 0.94 | 1  (1) | 0.69 | 1.5  (1) | 0.46 |
| one-day PE | 2  (0.5) |  | 1  (1.5) |  | 1  (0.25) |  | 1  (1) |  |

Table S2 - Detailed patient-reported outcome measure scoring comparisons. Note that 10 patients in the two-day/two-stage pelvic exenteration (PE) group completed their 12-month follow-up, and all 11 completed 6-month follow-up; with 7, 8, and 9 patients returning PROMs at 3-months, 6-months, and 12-months respectively in the one-day PE group. IQR = interquartile range.
